# Controlling Cell Functions and Fate with Surfaces and Hydrogels: The Role of Material Features in Cell Adhesion and Signal Transduction

**DOI:** 10.3390/gels2010012

**Published:** 2016-03-14

**Authors:** Maurizio Ventre, Paolo A. Netti

**Affiliations:** 1 Department of Chemical, Materials and Industrial Production Engineering and Interdisciplinary Research Centre on Biomaterials, University of Naples Federico II, P.le Tecchio 80, 80125 Napoli, Italy; maventre@unina.it; 2 Center for Advanced Biomaterials for Health Care@CRIB, Istituto Italiano di Tecnologia, L.go Barsanti e Matteucci 53, 80125 Napoli, Italy

**Keywords:** cell adhesion, surface patterning, hydrogel, mechanotransduction

## Abstract

In their natural environment, cells are constantly exposed to a cohort of biochemical and biophysical signals that govern their functions and fate. Therefore, materials for biomedical applications, either *in vivo* or *in vitro*, should provide a replica of the complex patterns of biological signals. Thus, the development of a novel class of biomaterials requires, on the one side, the understanding of the dynamic interactions occurring at the interface of cells and materials; on the other, it requires the development of technologies able to integrate multiple signals precisely organized in time and space. A large body of studies aimed at investigating the mechanisms underpinning cell-material interactions is mostly based on 2D systems. While these have been instrumental in shaping our understanding of the recognition of and reaction to material stimuli, they lack the ability to capture central features of the natural cellular environment, such as dimensionality, remodelling and degradability. In this work, we review the fundamental traits of material signal sensing and cell response. We then present relevant technologies and materials that enable fabricating systems able to control various aspects of cell behavior, and we highlight potential differences that arise from 2D and 3D settings.

## 1. Introduction

For a long time, cell-culturing substrates, like glass, plastic and metal, were considered as passive supports. In these systems, soluble biochemical supplements were regarded as key players in affecting cell fate and functions. However, a growing body of experimental evidence has come to light in the recent past and has clearly demonstrated that the chemical-physical properties of the scaffolding materials can be as effective as the soluble biochemical signals [1]. This should not be surprising, since each and every cell is constantly exposed to a multitude of signals *in vivo* that can be biochemical and biophysical in nature. In fact, cells can recognize and respond to mechanical forces of the surrounding environment, gradients of ligands and the topography of the tissues in which they reside [2]. Analogously, material substrates will invariably display signals to cells either deliberately or in an ‘unintentional’ manner. In other words, materials intrinsically possess their own stiffness, the distribution of adhesion signals; even what we consider a flat surface might display a topography at the nanoscale. Signals displayed by materials can influence a broad spectrum of cellular behaviors, such as adhesion spreading, migration, proliferation and differentiation [3,4]. Despite the sheer number of examples, only a few molecular mechanisms involved in the transduction of material stimuli in biological responses have recently been clarified [5,6,7]. This notwithstanding, a thorough understanding of the complex, molecular interplays occurring between material signals and cell response would bring in novel design concepts to engineer instructive materials able to control cell fate and functions in a deterministic manner. The practical benefits arising from such knowledge could be tremendous, since it can lead to the development of effective tissue-engineered products, tissue models to study development and pathologies *in vitro* and platforms for drug testing and discovery.

A large body of literature concerning the effects of material stimuli on cell behavior was focused on two-dimensional (2D) substrates that were instrumental in shaping our knowledge on the biochemical transduction of material signals. However, the effective translation of these findings in a clinical context requires the development of three-dimensional (3D) structures that better reproduce a physiological environment. In particular, tissue engineering and regenerative medicine failed in having a dramatic impact on modern clinics, despite their undeniable potentialities. This is mainly caused by a lack of knowledge on the effects of exogenous stimuli and in particular those presented by culturing materials, in the generation of fully-functional tissues *in vitro* or *in vivo*. This becomes particularly relevant in the case of stem cells that are very sensitive to micro-environmental signals [8]. In fact, signals presented to stem cells in their niche ultimately dictate fate and functions, *i.e.*, whether they have to remain quiescent, proliferate or differentiate [9]. In this context, one of the greatest challenges is to develop materials able to display a set of stimuli that tightly control stem cell behavior. This requires designing and fabricating perfectly-controlled physical/chemical environments in which the effect of specific material signals on cell functions can be precisely assessed. Developments in material science and related technologies, such as micro- and nano-fabrication and polymer functionalization, can be particularly useful to achieve this task. The modulation of a broad range of material features can be achieved in 2D setups with consolidated processes. However, tailoring the biochemical/biophysical characteristics of 3D environments requires much more sophisticated approaches. It has to be pointed out that the complexity in controlling cell behavior in 3D does not simply depend on the ‘added’ dimensionality. As will be soon clear, in 3D, cells perceive material signals differently from what happens in 2D. Furthermore, 3D substrates intrinsically possess additional features, not usually observed in 2D, like degradability or the possibility of undergoing extensive structural remodelling, which ultimately affect cell behavior. Hydrogels proved to be particularly useful in the context of cell behavior control through material features [10]. In fact, they possess chemical/physical characteristics that make them versatile platforms in which stiffness, porosity, bioactivity and degradability can be variously modulated.

In this work, we present and discuss some of the recent and most relevant findings concerning how material features can affect cell adhesion. We then analyze why modulating the adhesion event is important and how to achieve this with material patterning techniques. Finally, we provide examples on controlling cell functions and fate with specifically-engineered systems.

## 2. Mechanics of Cell Adhesion Formation on 2D or 3D Material Systems

Intuitively, the perception of material signals by cells requires some sort of contact followed by a probing phase. In fact, specialized molecular machineries are activated whenever the environmental conditions are permissive for a cell to adhere and spread on a substrate. More specifically, focal adhesion (FA) and the actomyosin cytoskeleton are the structures that play a fundamental role in adhering to and probing the extracellular environment [11,12]. They also provide the mechanical connection with which cells can exert forces to the ECM and *vice versa*: ECM transmits stress and strain to the cell cytoplasm. Several different types of macromolecules constitute FAs. Among these, integrins, transmembrane receptors, specifically engage ligands on the extracellular space, whereas proteins from the cytoplasmic side may exert a signaling (like focal adhesion kinase (FAK) and paxillin) or mechanical functions (like talin, vinculin, actinin and zyxin) [13]. Interestingly, the activity and dynamics of many adhesion molecules appear to be force dependent, for which contractile forces generated by the actin fibers can induce conformational changes that ultimately trigger signaling pathways [14,15]. The presence of certain ligands, the ways these are displayed by the extracellular space, along with their mobility are all factors that affect FA formation and maturation. Integrin clustering is an essential feature for the maturation of stable FAs [16]. Too few or sparse ligands might impair this process and halt the downstream signaling pathways [17]. Additionally, weakly-bound ligands or ligands tethered to flexible structures can be remodeled by the contractile forces exerted by the cell, and this can also affect cell response [18].

The concepts discussed so far are valid both *in vivo* and *in vitro*. In the latter case, materials need to be functionalized in order to display adhesive signals to cells. A broad range of chemical strategies and manipulation technologies have been developed and optimized so far in order to control cell adhesion events. Several works dating back to the early 1990s focused on modulating adhesion events affecting cell functions, such as spreading, migration and proliferation [19,20,21]. Diverse chemical functionalization strategies and fabrication technologies have been developed so far to precisely control the biochemical/biophysical features of the culturing substrate in order to direct cell behavior.

**Figure 1:**
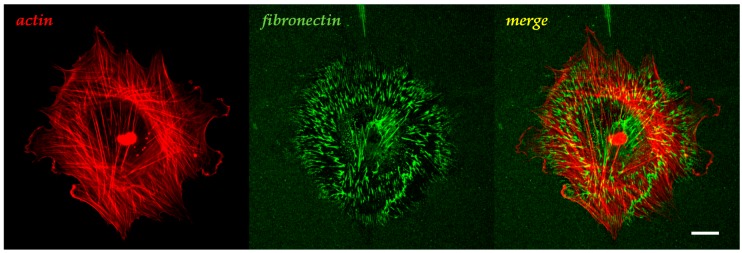
Confocal micrograph showing the effect of cell-generated forces on physisorbed fibronectin. MC3T3 preosteoblasts cultivated for 12 h on a nanograted, O2 plasma-treated PDMS substrate. Fibronectin (10 µm/mL) undergoes extensive remodelling caused by contractile forces. Fibronectin compaction is observed at both ends of actin fibers. Note how fibronectin smears follow the actin direction and leave a dark halo upon compaction. Actin is stained with Tetramethylrhodamine B isothiocyanate-phalloidin (red); fibronectin is stained by immunofluorescence (green). Scale bar: 20 µm.

Generally, the modulation of the cell adhesion events, and the cell response thereof, has been widely investigated in 2D setups. In this context, inorganic materials (glass, metallic alloys) or synthetic polymers (predominantly hard polystyrene (PS), polycaprolactone (PCL) or soft polydimethylsiloxane (PDMS), polyacrylamide (PAM)) have been largely used. Synthetic polymers proved to be particularly useful owing to their intrinsic versatility in allowing biochemical functionalization or the fine modulation of their mechanical properties in a broad range of stiffness. For instance, by simply changing the polymer/crosslink ratio, PAM hydrogels and PDMS elastomers can cover up to three orders of magnitude of Young’s modulus spanning from a few kPa up to MPa [22]. Additionally, many hard and rigid polymers are compatible with various micro- and nano-fabrication technologies, which allow embossing complex structures on their surfaces. Historically, adsorption of adhesive proteins (fibronectin, collagen, gelatin, vitronectin, laminin) has been routinely performed to make glass or synthetic materials bioactive. This however results in a poor control on ligand positioning and stability, and this becomes particularly relevant when a weakly-bound ligand layer experiences extensive cell-mediated traction forces. In this case, extensive ligand remodelling might occur, making it difficult to relate the cell response to the initial bioactive properties of the material surface (Figure 1) [23,24].

Furthermore, hydrophobic materials can denature physisorbed proteins, and this can affect the actual concentration of ligands presented to cells [25]. Covalent conjugation of proteins on synthetic materials allows gaining a better control over ligand stability and presentation. An enormous variety of chemical routes has been reported in the literature concerning the binding of biomolecules on surfaces, either with or without spacers. Most popular strategies involve glutaraldehyde, carbodiimide [26], sulfosuccinimidyl 6-(4′-azido-2′-nitrophenylamino)hexanoate (*i.e.*, sulfo-SANPAH) cross-linking [27] and the biotin-avidin binding system [28,29]. Yet, handling natural biomolecules to functionalize substrates can be expensive and time consuming. Furthermore, proteins can undergo denaturation or degradation as a result of the chemical treatments necessary for the coupling [30]. More recently, the use of peptide sequences that specifically interact with integrins has become a popular method to control cell adhesion on material surfaces or within scaffolds, owing to their increased stability towards chemical treatments. Examples of short peptide ligands include DGEA, RGD (derived from collagen), IKVAV, RGD, YIGSR (laminin), REDV and RGDS (fibronectin) [31]. RGD is certainly one of the most used and studied sequences, and several studies tracing back to the early 1990s investigated the density of RGD necessary to promote cell spreading and adhesion. Massia and Hubbell found that a density of 1 fmol/cm^2^ of RGD is sufficient for cell spreading on glass surfaces, whereas 10 fmol/cm^2^ are sufficient for focal contacts and stress fiber formation [19]. These figures strongly depend on the type of material substrate, since higher amounts of RGD peptides are generally required to achieve cell adhesion [32]. This seems to be related to the nature of the flexible linkers that connect the ligand to the surface; linkers might not provide the correct signal display or an effective mechanical feedback to cells upon contraction [33]. Furthermore, the chemical/physical properties of the surface may alter the effectiveness of ligand display. Additionally, the extracellular domain of integrins projects out of the membrane by ~10 nm, and it is likely that this is the maximum distance that allows for integrin-ligand engagement [34]. If the cell membrane cannot accommodate recesses on the material surface, then nanometric roughness on the material surface, or strata deeper than 10 nm in functionalized hydrogels, can in principle make ligands not readily accessible to the integrins.

While 2D setups possess undeniable advantages, like simple functionalization strategies, direct accessibility to the material regions to be functionalized, no resistance to nutrient transport and suitability to live examination with high magnification lenses, they cannot recapitulate the more physiologically-relevant, but complex 3D architectures found *in vivo*. The control of the biochemical/biophysical features of 3D environments requires the development and implementation of more complex processes. The 3D porous scaffolds used in tissue engineering applications are usually characterized by a pore size in the 100–500-mm range [35]. Within this range, cell behavior is affected by pore curvature [36]; additionally cells gradually fill up the pores and therefore do not perceive the same physical environment as the one sensed initially [37]. Nanofibrous electrospun mats might provide a microenvironment that is morphologically similar to native ECM; however, the modulation of the mechanical properties usually results in a modification of fibril diameter, pore size and bioactivity [38,39]. Conversely, polymeric and biopolymeric hydrogels not only provide cells with an *in vivo*-like 3D environment, but allow a fine tuning of the biochemical, microstructural and mechanical features through consolidated chemical/physical routes.

**Figure 2:**
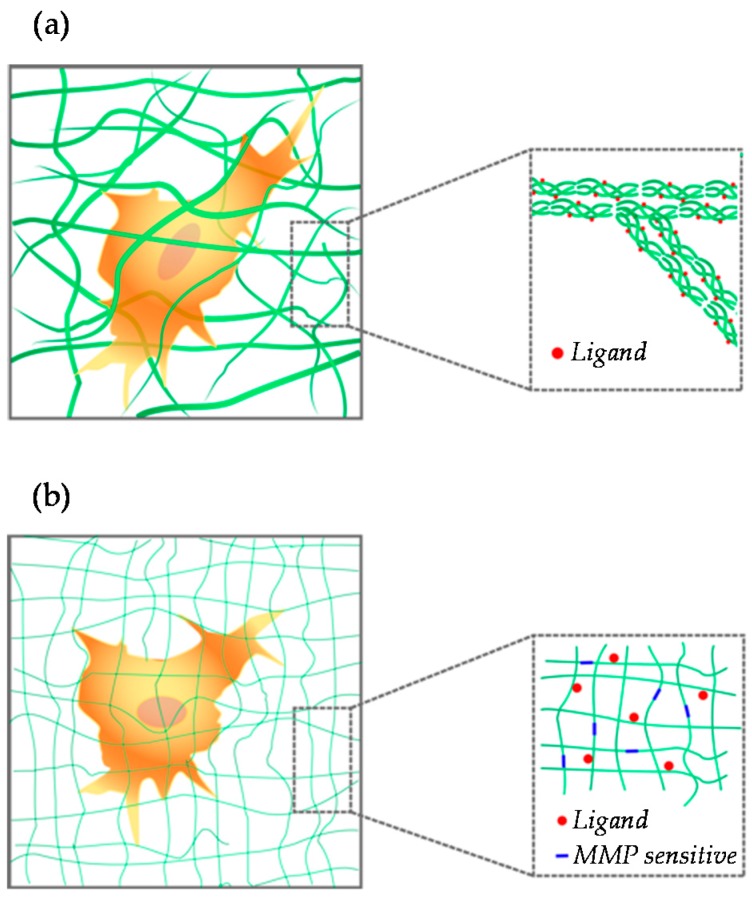
Schematic of cells encapsulated in hydrogels. (**a**) Natural fibrillar hydrogel: proteins self-assemble in the form of fibrils that form an entangled mass surrounding the cells. Fibrils constitutively display ligand motifs for cell attachment. (**b**) Polymeric hydrogel (synthetic or saccharidic): ligands need to be conjugated to the polymeric backbone, as well as degradable domains, to allow cells to adhere and spread.

Early examples of the use of hydrogels in cell biology concern fibrillar natural gels as collagen and fibrin. These are constituted by polypeptides that self-assemble in the form of microfibrils that intertwine in a 3D network (Figure 2a).

The gelation process occurs in mild conditions, thus allowing direct encapsulation of cells. Fibrils naturally display ligands for cell adhesion; therefore, additional functionalizations are generally not required. Despite these positive characteristics, natural fibrillar hydrogels are very compliant, and their structural mechanical and bioactive properties cannot be modulated independently. For instance, increasing the protein concentration results in an increase of gel stiffness, but adhesivity and porosity are also affected. This makes it difficult to assess the role of a specific hydrogel feature on the observed cellular response. However, the increase of modulus obtainable in this manner is marginal. Chemical crosslinking with glutaraldehyde has also been frequently applied, but unreacted molecules are extremely toxic. Other methods involving non-enzymatic glycation or genipin were used to effectively crosslink gels with minimal cytotoxic effects [40,41]. Matrigel is another natural hydrogel widely used for cell cultures. Its components, primarily laminin and collagen IV, are extracted from Engelbreth-Holm-Swarm mouse tumors [42]. Cells cultivated on or within Matrigel are able to recapitulate some morphogenetic processes that eventually lead to self-organized systems displaying striking similarity to native tissues and organs [43]. Also for Matrigel, the mechanical and structural properties cannot be tuned straightaway, and owing to its natural origin, batch-to-batch variability could occur.

Hydrogels whose constituents possess a “simple” chemical structure that can be precisely modified are steadily gaining popularity as 3D ECM analogues. These gels can be either of natural (agarose, alginate, hyaluronan) or synthetic origin (polyethylene glycol (PEG), poly(vinyl alcohol) (PVA), PAM). Basically, these materials form a sort of inert background, yet they possess an adequate number of groups to which selected functionalities can be added (Figure 2b). This kind of material represents valuable and versatile tools whose chemical/physical features can be independently modulated to a large extent. Natural fibrillar hydrogels, like collagen, fibrin or gelatin hydrogels, are endowed with ligands to which cells can adhere. Conversely, synthetic and polysaccharide hydrogels have to be modified in order to correctly display binding sites. This can be achieved by conjugating short peptide sequences or biomacromolecules (collagen, fibronectin) to the polymer backbone.

While the mechanisms on cell adhesion in 2D setups have been extensively characterized, the composition and dynamics of cell binding in a 3D environment are less defined. In fibrillar collagen gels, proteins, such as vinculin, paxillin, zyxin and talin, were found [44,45]. Additionally, while FA length correlates with substrate stiffness in 2D, long FAs are observed in 3D soft matrices provided that fibrils are coaligned with the FA axis. Furthermore, the level of zyxin and vinculin correlated with FA size [46]. Taken together, these data depict an intricate scenario in which adhesion dynamics, composition and morphology in 3D are affected by multiple chemical/physical features of the microenvironment, and the extrapolation of 2D results in a 3D context is not straightforward. This notwithstanding, there is growing evidence that adhesion molecules play an important role in 3D mechanotransduction in a similar manner as in 2D setups.

## 3. Engineering Materials to Control Cell Fate and Functions

### 3.1. Surface Patterning

Cells adhere to surfaces through a limited and well-defined number of points, *i.e.*, focal adhesions. Even in the case of surfaces uniformly coated with ligands, adhesion occurs in discrete locations. In fact, the maturation of adhesion involves the recruitment of transmembrane and cytoplasmic molecules at the site of adhesion. If this process is halted, for instance by too few or sparse ligands, adhesions can disassemble. Therefore, the presence/absence of the ligand is not the only parameter that affects adhesion events, but it is rather the way the ligand is presented that plays a non-negligible role. For instance, ligands’ mobility (through flexible tethers), their density, spatial positioning, along with substrate stiffness and topography are all factors that eventually influence FA establishment, maturation and activation of signaling pathways consequently. Evidence of this was provided by Maheshwari *et al.*, who engineered PEG star-based polymeric substrates displaying YGRGD ligands in a clustered form, *i.e.*, 1, 5 or 9 ligands per star. Additionally, the authors were able to control the intercluster spacing that varied from 6–300 nm and achieved RGD surface densities in the 0.9 × 10^3^–1.2 × 10^5^ ligand/cm^2^ range [47]. NR6 fibroblasts displayed increased spreading and speed when the ligand was presented in a clustered form with respect to what was observed on non-clustered arrangements. Interestingly, a minimum cluster distance of 60 nm was required to permit adhesion and stress fiber formation, whereas 6 nm was calculated to be the threshold value for non-clustered ligands. These data point out that both ligand clustering and interligand spacing are important parameters in affecting cell adhesion.

The high sensitivity cells possess in recognizing ligand arrangements is not limited to differences in density or clustering. For instance, gradients of signals provide cells directional information necessary for polarization and migration, and various gradients of either bound or soluble signals are found *in vivo* [48,49].

A broad spectrum of methods was developed to generate concentration gradients of ligands on synthetic substrates. Methods based on plasma or light irradiation, diffusion, microcontact printing (µCP) and microfluidic, reviewed in Wu *et al.* [50], proved to be effective in generating gradients of ligands and enabled a precise control on gradient slope and average concentration. Combining photochemical and electrochemical approaches, Lee *et al.* fabricated RGD gradients on electroresponsive SAMs [51]. The authors studied the migratory response of 3T3 fibroblasts on different gradient slopes. Fibroblasts were very sensitive to both local ligand density and slope. In fact, cells on steep gradients terminated their migration in regions with a higher local RGD concentration with respect to cells migrating on shallow gradients. Furthermore, the authors showed the importance of FAK in sensing ligand presentation, as knockout FAK cells positioned themselves to the same density irrespective of the gradient slope. Concerning migration speed, Smith *et al.* used a diffusion-based method to realize fibronectin gradients on SAMs [52]. Endothelial cells showed a drift speed that correlated with gradient slope, whereas the random component of speed, along with the persistence time remained constant. Possibly, this behavior may arise from higher frequencies of cell polarization or its increased stability at higher gradients. Analogous results were obtained by Guarnieri *et al.*, who analyzed NIH/3T3 migration atop PEG hydrogels with RGD gradients fabricated with a fluidic gradient generator [53]. The authors found increased cell alignment on steep gradients. Drift speed correlated with gradient slope up to a threshold value for very steep gradients. These data, along with others, clearly demonstrated that specifically-engineered platforms displaying continuous gradients of ligands in biologically-relevant concentrations represent a valuable tool not only to unravel the basic mechanism underlying cell response to signals in a density-dependent manner, but also to effectively define the optimal concentration of bound signals for a specific cell response [54].

The methods described above, while instrumental in shaping our understanding of signal recognition and cell response, are not able to provide an accurate control of ligand spatial positioning on a micro- to nano-metric scale. This is a central aspect to create perfectly controlled environments for performing systematic studies on cell adhesion events. The implementation of micro- and nano-fabrication technologies allowed functionalizing material surfaces with a high spatial resolution and with reasonable costs and processing times. Photolithographic techniques are probably the cornerstone of all of the surface functionalization technologies aimed at fabricating functional surfaces to control cell adhesion. Photolithography consists of the exposure of a substrate, typically silicon, coated with light-sensitive photoresist with a patterned UV light. Light patterning is most conveniently performed by applying a specifically-designed reflective mask. In case of ‘positive’ photoresists, only those parts exposed to the radiation are soluble in organic solvents. Conversely, in ‘negative’ photoresists, the solvent dissolves the non-exposed parts, thus creating an inverse pattern. This process requires specialized equipment and high capital costs. However, the technology is nowadays very well consolidated, and raw materials are easily affordable: therefore, the fabrication of patterned surfaces can be outsourced. This allowed the development of soft lithographic techniques in which an elastomeric stamp or master, usually in PDMS, is fabricated and employed to create patterned surfaces. Patterned stamps can be treated with oxygen plasma to improve hydrophilicity and/or can be coated with proteins to promote cell adhesion and then can be used directly as substrates for cell cultures.

Thus defined, soft lithographic techniques encompass a broad spectrum of processes, among which replica molding (REM), µCP and micromolding in capillaries (MIMIC) have been extensively used to confine cell adhesion sites with a micrometric or sub-micrometric spatial resolution [55,56]. REM of synthetic polymers consists of embossing the topographic features of the elastomeric stamp onto polymer precursors or melted polymers, which are then solidified. Patterned elastomeric masters or structures fabricated via REM have been largely used to assess the effects of topographic features on cell behavior. Systematic studies on this issues regarded patterns in the form of gratings, pillars and protrusions. Basically, these substrates display ‘terraces’ on which cells can form adhesions, juxtaposed to recesses that might not be readily accessible. Topographic patterns having too narrow and/or too deep features might not allow the cell membrane to accommodate surface contours, thus causing the cells to be ‘suspended’ on the top of ridges and pillars. Furthermore, if these structures have lateral sizes, which may interfere with the normal formation and maturation of FAs, then alterations in cell adhesion, spreading orientation and migration are observed. In the case of parallel nanogratings, FAs and stress fibers are predominantly oriented along the pattern direction. In this circumstance, most of the cell types exhibit an elongated morphology and migrate preferentially parallel to the pattern direction [57]. This phenomenon, usually referred to as contact guidance, ceases to exist when topographic features are so shallow to not be recognized by the cell anymore. Apparently, the threshold depth below which features do not exert their regulatory role on migration and alignment is 35 nm [58].

To gain a better insight into the origin and effect of contact guidance on nanopatterned substrates, we used fluorescent tags to investigate the dynamics of FAs and cytoskeleton assemblies [59]. We found that actin fibers with directions different from that of the pattern possessed dashed adhesions that colocalized in the proximity of consecutive ridges. Such a peculiar assembly was caused by the confining effect induced by the nanopattern on FA growth. FAs thus formed were unstable and rapidly collapsed under the effect of actin-generated forces. Eventually, the vast majority of FAs were coaligned with the pattern direction, which in turn affected cytoskeletal structure and cell shape.

Micron- and submicron-scale patterns were also shown to affect cell proliferation [60,61]. However, literature studies are not conclusive on this aspect, as it seems that no obvious trends exist that allow one to predict the effects of topographic patterns on proliferation [62].

µCP uses the elastomeric master as a stamp to transfer molecules or proteins on surfaces. Usually, the surfaces are composed of or coated by a cell-repellent material, for example a self-assembled monolayer (SAM) of PEG. In this case, a sharp mismatch in adhesion properties is made, which results in the confinement of cell adhesion. Csucs *et al.* transferred patterns, with lateral resolution down to 1 µm, of adhesive molecules (either peptides or proteins) on various materials [63]. Adhesion mismatch was induced by poly-l-lysine-g-PEG backfill. This work demonstrated that through a careful optimization of the material properties and patterning procedure, the pattern was made very stable, even in the presence of serum proteins, which might in principle alter the ligand distribution on the surface. In fact, cells adhered on the functionalized regions only, and a strong directional confinement was observed during cell migration. More recently, Eichinger *et al.* proposed the development of the conventional µCP technique for multi-molecule transfer [64]. The development involves the use of modified inverted microscopes for proper stamp alignment prior to printing. The authors fabricated alternating micro-stripes of laminin and aggrecan and showed that astrocytes correctly recognized the multi-molecular pattern and adhered onto the laminin stripes only. This example extends the range of potential applications of µCP in settings requiring complex multimolecular patterns.

In MIMIC, a patterned elastomeric stamp with an open network of channels is pressed against the surface that needs to be functionalized. A solution containing the ‘functionalizing’ molecule is delivered through the network by capillary suction. The solution can be composed of polymer precursors or proteins. Solutes in the fluid can then be adsorbed on one surface or can be treated chemically or thermally, thus replicating the pattern features of the network. This method proved to be straightforward and effective in confining cell adhesion at a single [65] or multiple cell level [66].

With the above-mentioned methods, the size of pattern features displayed by the elastomeric stamp is limited by the diffraction of the UV light. Submicron-scale features can be obtained by using, for example, extreme UV light. Methods aimed at challenging the diffraction limit of light, such as electron beam lithography (EBL) and focused ion beam lithography (FIB), were developed in order to fabricate nanoscale features. These techniques use short wavelength electromagnetic sources and do not require a mask, as the beam is deflected on the surface with electromagnetic lenses. Additionally, FIB allows atoms to be displaced from or deposited onto the material surface, in which case it is possible to achieve subtractive or additive lithography on the final substrate directly, without further development. These techniques acquire particular importance when a spatial control on single integrin clusters or even individual ligands are required.

Using EBL combined with imprinting lithography, Schvartzman *et al.* fabricated arrays of metallic clusters constituted by AuPd nanodots assembled in different arrangements from dimmers up to heptamers. Each dot displayed a single RGD ligand, thus exerting a remarkable control on adhesion events at a single molecule level. The authors found that the overall density of dots did not affect spreading dramatically, whereas cluster size was crucial. Furthermore, tetrameric clusters of ligands, with interligand spacing of 60 nm, were necessary to enable cell spreading [67].

The fabrication of patterned surfaces exhibiting extremely small features is achieved at the expense of the processing time, which may render these nanotechnologies not particularly suitable when large-area patterning is required. A partial solution to this issue is represented by micellar lithography that involves the spontaneous arrangement of polymeric micelles, with a nanometric metal core, in a closely-packed quasi-hexagonal lattice on material surfaces [68]. For a careful modulation of the processing conditions and micelle characteristics, patterns with spacing ranging from 28–85 nm were reported. Metallic nanoparticles can be decorated with ligands or other biomolecules; therefore, by controlling the particle size and interparticle spacing, a tight control over FA formation and maturation can be achieved. By exploiting this technique, Arnold *et al.* fabricated quasi-hexagonal patterns of RGD functionalized gold nanodots with ligand spacings of 28, 58, 73 or 85 nm [69]. The authors observed a sharp transition of cell response in terms of adhesion and spreading passing from 58–73 nm. In particular, ligand spacing above 73 nm did not favor FA formation and actin assembly. Therefore, a ligand spacing of 58 nm is necessary to permit integrin clustering, thus triggering the cascade of events that lead to adhesion formation. Follow-up studies involving substrates displaying gradients of nanodot spacing (50–250 nm) found that a spacing slope of 15 nm/mm is the minimal slope required to induce cell polarization and demonstrated the exquisite sensitivity of cells in recognizing small spatial variations in ligand separation (~1 nm) [70]. These data, together with those of Maheshwari and Schvartzman, indicate that ~60 nm is a characteristic ligand distance above which adhesion formation is impaired. It was suggested that such a distance is required for talin binding, which then stabilizes integrin clustering [67]. While these figures seem to be consistent among various anchorage-dependent cell types, maximum interligand spacing of 32 nm was shown to be necessary for hematopoietic stem cells to adhere [71]. In fact, above such a threshold value, integrin clustering and lipid raft-dependent integrin signal transduction were strongly depressed.

### 3.2. Hydrogel Engineering

A large body of literature has been produced in the past few decades on hydrogel engineering to specifically control cell functions. These include cell adhesion, migration, spreading and differentiation. To this aim, hydrogels must be endowed with specific biological activities and microstructural features. First, cells have to be encapsulated within the hydrogel. Second, whatever chemical functionalization is chosen, this should not harm cells during the encapsulation process nor in culture. Concerning encapsulation, cells are usually suspended in a prepolymer solution. Gelation can be promoted by the insertion of reactive crosslinking molecules or it might rely upon physical forces. Various chemical crosslinking schemes have been developed, which proved to be cytocompatible and do not affect hydrogel bioactivity. These schemes include radical polymerization, click reactions and Schiff base crosslinking, reviewed in [72,73]. Usually, to gain a better spatial and temporal control on the reaction process, light-sensitive molecules are employed, which generate radicals upon exposure to the adequate radiation. Various combinations of polymers-photo initiators have been proposed so far [74]. However, some concerns have been raised on the toxicity of the photo-initiator and the UV radiation, which prompted the development of cell-friendly photo-crosslinkers [75].

Natural fibrillar hydrogels intrinsically possess peptide sequences that promote cell adhesion; they do not possess the adequate versatility in tuning the biochemical, structural and mechanical features independently, which is a fundamental requisite to exert a tight control on cell adhesion and response. Conversely, synthetic and polysaccharide hydrogels are characterized by chemical structures that make them more prone to achieving an orthogonal control on their biochemical/biophysical properties.

Different strategies can be pursued to engineer the biochemical/biophysical properties of hydrogels. These can be roughly grouped into top-down or bottom-up approaches. The former consists of chemical strategies that are optimized to conjugate active molecules or sequences to structural, possibly inert backbones, mainly of synthetic origin, thus creating a hybrid system. By exploiting this approach, Lutolf *et al.* formulated a PEG-based hydrogel containing both RGD- and MMP-sensitive domains [76]. This allows an orthogonal control on cell adhesion and cell-mediated degradation, which are elements of paramount importance to systematically investigate cell behavior *in vitro* or *in vivo*. Fibroblasts were able to break down the matrix by expressing MMP. Furthermore, their response, in terms of invasion, to ligand concentration was similar to what was observed in 2D setups, with cells displaying a biphasic dependence of migration on ligand density [77]. Finally, the authors proved the effectiveness of the hydrogel system in promoting bone regeneration in a critical size defect model. Taken together, this landmark study demonstrated that specifically-engineered hydrogels possess an enormous flexibility and versatility in the design of the material properties. This allows developing not only valuable tools to investigate complex cell-matrix interactions, but also systems that exert a therapeutic potential *in vivo*.

Hybrid systems were also designed in order to exploit peptide crosslinks, which form in mild conditions. For instance, Sanborn *et al.* fabricated a four-arm PEG functionalized with a fibrin-mimetic peptide that crosslinks in the presence of calcium divalent ions, thrombin and factor XIII, thus generating an elastic gel at 37 °C [78]. More recently, Ehrbar *et al.* followed a similar approach by functionalizing PEG macromers with specific substrate peptides that induced gel formation in the presence of Ca++ and factor XIII [79]. The use of peptide-synthetic hybrids is beneficial not only for modulating the hydrogel features, but it also allows encapsulated cells to perform complex functions. In the example above, human fibroblasts were able to cleave the peptide sequences of the network through matrix metalloproteinases (MMPs), which ultimately resulted in network remodelling with the gel forming extensive dendrite-like connections.

Many other biologically-derived features were implemented in synthetic hydrogels. For instance, the natural mechanism of growth factor (GF) sequestration/release via ECM binding molecules was implemented in synthetic hydrogels by means of different techniques [80,81]. On-demand release of active GF was achieved by Zisch *et al.*, who developed a PEG-based hydrogel containing both RGD for cell adhesion and VEGF coupled with MMP-sensitive domains [82]. This system has the advantage of avoiding systemic release of factors and limits its activity only upon cell-mediated proteolytic remodelling. This and other examples discussed in the following section demonstrate once again the superior capabilities of synthetic hydrogels in controlling the development of complex biological processes thanks to their ability to enable an orthogonal modulation of mechanical, biochemical and structural properties.

Top-down approaches, while beneficial for the realization of 3D macroscopic systems with clearly-defined biological properties, might not allow for accurate spatial arrangement of the bioactive, functional or structural elements. If spatial control of signals’ display is desired, bottom-up approaches are more adequate. For instance, promoting or disrupting crosslinks in specific locations with patterns of light proved to be effective in creating hydrogels with well-defined microstructural features down to the sub-micrometric range.

Patterning of biochemical, mechanical, topographic signals can be achieved in 2D with consolidated and widespread techniques. Hydrogels, or more generally, 3D patterning, pose non-trivial technical hurdles. Patterned polymeric stamps have been used to confine hydrogel shape during gelation, thus creating simple networks of channels. By using such a method, Nelson *et al.* generated branched micro-channels in collagen gels and reported that their geometry can control epithelial morphogenesis by dictating the local microenvironment [83]. This method, although simple and robust, cannot be employed for the fabrication of complex 3D architectures. This notwithstanding, several processes have been developed that are able to locally manipulate gel structure or chemistry in a consistent and effective manner. In this part, we will present a limited number of hydrogel patterning technologies that were effective in spatially arranging signals, thus affecting cell behavior. Comprehensive reviews on 3D pattering technologies can be found in the specialized literature [84,85]. Basically, the technologies can be divided into two macro-sectors: spatial control of the gel crosslinking and spatially-controlled deposition of materials (that usually gels immediately after deposition on a supporting material or structure). The first category is dominated by light-induced crosslinking or dissolution. Stereolithographic methods rely on focusing of a light beam in a bath containing light-sensitive molecules that enable gel formation upon exposure to light. To produce 3D macroscopic objects, the fabrication occurs in a layer-by-layer fashion. Different approaches can be pursued to achieve the desired 3D structure with a 1–10-µm spatial resolution. In one approach, a fabrication platform is located beneath the prepolymer liquid interface and moves downwards as the hydrogel layers are inscribed on the platform top. Alternatively, the fabrication starts with the platform close to the prepolymer bath bottom (made of a transparent glass). As the hydrogel layer is formed by the light beam, the fabrication platform moves upwards, carrying along the newly-formed hydrogel structure. Platform movement is controlled by motors, which must allow for the micrometric motions, and the step between two adjacent layers is smaller than the curing depth produced by the light beam. The light source is generally a laser whose beam path is controlled by micro-tilting mirrors or stages. More recently, digital mirror devices or LCD screens have been employed to project light patterns on or beneath the prepolymer bath [86,87].

These techniques have been used to fabricate hydrogels with predefined ordered structures by employing either synthetic (poly(2-hydroxyethyl methacrylate) or PEG-diacrylate [88,89]) or natural (gelatin or alginate [90,91]) materials.

As previously discussed, the spatial resolution of hydrogel features is limited by the diffraction of light. Ten micrometer-wide features can be easily achieved. To improve spatial resolution, thus fabricating narrower features, different writing technologies have been developed. Along this line, multiphoton microscopy proved to be effective for hydrogel patterning. In fact, the focal point of femtosecond near-IR light is used to initiate the crosslinking reaction in a very small volume, leaving the rest of the prepolymer unaffected. By moving the focal point in the space, it is possible to fabricate complex structures with sub-micrometric resolution. Also for this technique, synthetic (PEG-diacrylate [92]) or natural (gelatin [93]) natural materials can be used.

Careful optimization of the processing conditions and crosslinking mechanism allows encapsulating cells within the patterned gel during fabrication, directly. For instance, Chan *et al.* investigated the viability of NIH-3T3 cells encapsulated in PEG hydrogels patterned with stereolithographic methods [94]. According to the processing conditions, the authors reported good cell viability and homogeneous seeding after seven days of culture. These data demonstrate the feasibility of patterning cell-hydrogel hybrids *in situ* with a high spatial resolution.

Material deposition-based approaches require the materials in the form of prepolymer to be extruded through a dye. Crosslinking occurs immediately after deposition on a supporting material with the gelation process being driven by external stimuli. For instance, Tirella *et al.* were able to fabricate honey-comb-like structures of alginate, crosslinked upon exposure to a CaCl_2_ solution using a pressure-assisted micro-syringe technique [95]. The authors achieved hydrogel feature sizes down to 200 µm. A similar technology is represented by 3D-bioplotting; in this case, the prepolymer is extruded in a coagulation bath in which gelation might occur via temperature changes or chemical reactions induced by the medium. Generally, 3D-bioplotting allows the gelation to occur in mild conditions, which is amenable for direct cell encapsulation [96].

The fabrication of a fine network of hollow channels was made possible by subtractive methods. Recently, Miller *et al.* exploited a plotting-based technique to create an ordered network of micron-scale capillaries within a collagen gel [97]. Briefly, carbohydrate glass fibers, with diameters down to 200 µm, were plotted in a regular 3D lattice. Hydrogel precursors (either agarose, alginate, fibrin, Matrigel or PEG) were poured onto the network, allowing the solution to fill the porosity. Dissolution of the fibers generated a hollow and interconnected regular porous network. The authors fabricated a perfusable network of micro-vessels, lined with endothelial cells, surrounded by a cell containing gel, thus generating a vascularized tissue mimic *in vitro*. Subtractive patterning was also achieved by exploiting thermal degradation of materials. Using a near-infrared femtosecond laser, Hribar *et al.* controlled the thermal denaturation of cell-populated collagen-gold nanorod composites, thus creating channels with diameters down to ~8 µm, whilst maintaining good cell viability [98].

Spatial control of laser light was also employed to precisely locate biological functions of hydrogels, thus exerting a localized control of cell response. Kloxin *et al.* synthesized photodegradable PEG hydrogels’ remote manipulation of gel properties *in situ* with UV irradiation of a two-photon laser [99]. Dissociation occurred upon irradiation, which resulted in the formation of channels that confined cell migration. The same concept was used to locally alter biochemical properties by conjugating bioactive moieties, here RGD, with photolabile sequences. Follow-up studies further demonstrated that the technology allows for a precise and predictable tuning of the gel structure in real time, which results in the control of the behavior of individual cells [100]. Lee *et al.* used laser light to generate patterns of RGD ligand within collagenase-sensitive PEG hydrogels [101]. RGD ligands covalently bound to the pre-gelled PEG network in the regions irradiated by the laser beam only. Human fibroblasts showed guided 3D migration only into the RGD-patterned regions of the hydrogels.

Sophisticated 3D-bioplotters equipped with multi-nozzle lines to deliver multiple material types have also been developed in order to fabricate organotypic cultures *in vitro*. Lee *et al.* assembled a pneumatic-driven four-channel plotter able to extrude collagen fibers or cells in a coagulation bath, thus creating a multi-layered skin equivalent constituted by dermis and epidermis strata [102]. Concerning lateral resolution, the definition of a limiting feature size depends on several parameters, such as dye shape, extrusion process, crosslinking method and material type. This stated, a broad range of lateral resolutions has been reported in the literature [103] spanning from a few up to hundreds of microns.

Recently, printing-based approaches have been extended to hydrogel materials. While the technology is more suitable for solid materials, specifically-designed apparatuses allowed the fabrication of patterned 3D structures with micron-scale resolution. Lam *et al.* printed powders of a polysaccharide gelatin blend that were bound together by water [104]. The use of water as a binding agent possesses the advantage of enabling material gelation in mild and cytocompatible conditions, in which the use of labile factors and biomolecules can be envisaged. An issue might arise on the mechanical strength of the final product; therefore, post-processing reinforcements are usually required. Similarly, Xu *et al.* fabricated a multi-layered fibrin hydrogel in the form of a fibrillar matt for *in vitro* neuron cultures [105]. The authors printed thrombin solution (crosslinking agent) over a layer of fibrinogen (fibrin precursor). Upon gelation, neurons were printed on the fibrillar hydrogel. The operation was reiterated five times, thus producing a 3D scaffold. Additionally, the authors reported the maintenance of phenotypic electrophysiological fingerprints for neurons seeded in 3D.

Advancements in biomaterial synthesis and plotting technologies allowed the development of bioprinting-based techniques in which ”bioinks” made of cells and supporting materials are delivered in an orderly on a scaffolding “biopaper”. The main component of the bioink is represented by cells, which are preliminarily assembled in the form of spheroids or cylinders. These are plotted on an inert hydrogel, the biopaper in the desired shape. Additional hydrogel struts might be required to generate complex structures, such as hollow cylinders. Then, the process is reiterated in a layer-by-layer fashion. Printed tissues/organs are cultivated in incubators, and the spheroids fuse together. The structure then matures, undergoing morphogenetic events reminiscent of early embryonic development. In this process, the presence of a hydrogel is crucial, as it provides a supporting frame that dictates the final shape of the construct. In particular, when different cell types are used to form the bioink particles, a segregation of cells occurs, forming cellular patterns observed in natural tissues. This phenomenon was exploited to generate complex tissues, such as blood vessels [106], cardiac tissue [107] and nerves [108].

It should be pointed out that many of the hydrogel patterning technologies described above were predominantly aimed at tissue engineering applications or at guiding the behavior of cell populations. Current limitations of the 3D patterning technologies do not allow exerting a fine control on the spatial positioning of ligands or topographic features at the nanoscale, which is necessary to control adhesion processes at a single cell level. However, the steady advancements in optical methods, for example two-photon microscopy, hold the promise to enable 3D hydrogel pattering with a nanometric resolution and in cytocompatible conditions.

## 4. Mechanosensing and Mechanotransduction

The functions of FAs are not limited to cell adhesion. Many signaling proteins, such as FAK, ERK, JNK and Src, are contained within FAs and are involved in a broad spectrum of signaling pathways that regulate diverse aspects of cell behavior, such as migration, proliferation and differentiation [109]. Therefore, besides their mere structural functions, FAs are important signaling centers. Since FAs’ shape, composition and dynamics are very sensitive to exogenous stimuli, it is in principle possible to control complex cellular functions through the modulation of FA formation and growth. The examples described above clearly demonstrate that ligand presentation, in terms of spatial density and clustering, is an effective manner to modulate FA dynamics. Moreover, since many molecules participating in FA formation are mechanosensitive, change their activity and interact differently with other partners if subjected to actin-generated forces, the manipulation of cell contractility via material stiffness also affects adhesion-related signaling pathways and eventually cell functions. In fact, there is growing evidence that these approaches, *i.e.*, controlling ligand density, availability and positioning through biochemical or topographic patterns, or modulating FA dynamics with material stiffness, lead to strikingly similar effects on cell functions and fate.

Cell sensing of and response to material stiffness has been traditionally studied on synthetic hydrogels. For instance, PAM hydrogels were employed to study cell adhesion, migration and differentiation in experimental conditions in which stiffness could be varied in ranges similar to those measured in natural tissues. Our current understanding of mechanosensing and mechanotransduction is predominantly based on 2D setups, and yet, many biological mechanisms still need to be thoroughly elucidated. This notwithstanding, it is now widely accepted that adhesion formation and stress fiber contraction play a central role in mechanosensing [110]. In this process, the way ligands are anchored to the substrate, for instance either with flexible or rigid tethers, is crucial. More specifically, when cells pull the substrate, the actual stiffness they perceive is an integrated mechanical property in which both material elasticity and ligand mobility play a role. Concerning the latter aspect, Houseman *et al.* conjugated RGD on glass by means of oligo(ethylene glycol) spacers of various lengths [111]. The authors found that for a fixed density of peptide, increasing the length of the spacer significantly decreases the efficiency of cell attachment and spreading. Conversely, Kuhlman *et al.* developed a poly(methyl methacrylate)-graft-poly(ethylene oxide) system in which PEO segments of different lengths displayed RGD [112]. The authors found that the longer tethers increased the rate of fibroblast spreading and reduced the time for FA formation. Presumably, the tether flexibility favored integrin clustering and FA formation. This apparent discrepancy between the findings might arise from the differences in the range of tether lengths and ligand densities that were investigated. However, these data demonstrated the high sensitivity that cells possess in recognizing and reorganizing signals at the nanoscale. Along this line, Choi *et al.* have recently reported the development of a facile platform to study the influence of RGD ligand coupling strength on hMSCs’ spreading and differentiation, while maintaining substrate hydrophilicity and ligand density constant [113]. The authors reported enhanced osteogenesis and Yes-associated protein activity on tightly-bound RGD substrates that confirm the importance of ligand elasticity in dictating stem cell fate.

This notwithstanding, the regulatory role of bound signal stretching on cell fate and functions has not been thoroughly understood. Trappmann *et al.* highlighted that ligand tethering can be influenced by substrate stiffness [114]. In fact, the authors found that stiffer PAM hydrogels possess a smaller mesh size that offers more anchoring points for bioadhesive molecules. This results in an enhanced mechanical feedback. More recently, Wen *et al.* used an analogous material platform, namely PA gels, to study MSC response to changes in substrate stiffness, porosity and ligand tethering [115]. Differently from the previous work, the authors demonstrated that the main regulator of MSC fate was the substrate bulk stiffness, whereas porosity and tethering had negligible effects on cell fate. Other platforms were developed and presented in the literature that allow tuning substrate stiffness and adhesivity independently. For instance, Fu *et al.* developed arrays of microposts in PDMS on top of which cells were suspended [116]. Post deflection was observed upon cell contraction. With this setting, the bending stiffness could be modulated by changing the post height, while keeping adhesive properties unchanged.

The transduction of mechanical signals into biological events gained attention when Engler *et al.* showed that stem cell lineage specification was driven by PAM elasticity, with MSCs differentiating into neurons, myoblasts or osteoblasts when the stiffness of the underlying matrix approached the stiffness of brain, muscle or bone, respectively [117]. Stiff gels induced a more contractile phenotype and promoted osteogenesis, whereas less spread and contractile cells differentiated into adipocytes. Other studies, not directly exploiting matrix stiffness, but aimed at manipulating cell contractility through cell shape, reported similar observations, *i.e.*, shapes promoting myosin-driven contractility-enhanced osteogenesis, whereas round shapes induced little cell contractility and promoted adipogenesis [118,119,120]. This viewpoint has been recently challenged by Wang *et al.*, who observed increased osteogenesis and adipogenesis on RGD-functionalized gold nanodot patterns, which in principle should depress cell spreading, thus favoring adipogenesis [121]. Even though the underlying mechanisms have not been thoroughly elucidated, the authors suggested that RGD nanospacing might be an inherent signal that regulates differentiation of stem cells beyond cell spreading. Since the structure and stiffness of natural tissues are the result of continuous remodelling and biosynthetic processes that take place both in morphogenesis and during tissue homeostasis, it is not surprising that cells might respond differently if subjected to time changing material stiffness with respect to static materials. This aspect was investigated by Young and Engler [122], who cultivated cardiomyocytes on HA-PEG hydrogel that was subjected to a crosslinking program aimed at recapitulating the stiffening of native cardiac ECM during morphogenesis. The authors found that increasing matrix stiffness according to biologically-inspired time programs improved cardiomyocyte differentiation.

Most of the works dealing with the influence of substrate stiffness on cell adhesion are based on studies performed on single material types, chiefly PAM, PEG and PDMS. Therefore, possible effects arising from the chemistry of the material rather than its intrinsic mechanical properties might not emerge clearly. Additionally, substrate materials are usually treated as linear elastic materials. However, soft hydrogels and elastomers, as those used in mechanobiology studies, are viscoelastic in nature. Thus, the hypothesis of linear elasticity can be misleading when drawing out general conclusions on the role of material mechanical properties on cell behavior, especially when time-dependent effects are taken into consideration. The role of substrate viscoelasticity in altering cell mechanotransduction was carefully addressed by Cameron *et al.* [123]. The authors cultivated human mesenchymal stem cells (MSCs) on different PAM hydrogels, having constant stiffness (elastic modulus), but varying levels of creep (loss moduli). A decreased size and maturity of FAs and an increased proliferation, spreading and differentiation towards multiple lineages, with a propensity towards myogenesis, were observed for cells on high-creep hydrogels. The authors suggested that the decrease in isometric tension (*i.e*., contractility) is compensated by an increase in isotonic tension, which favors spreading and more dynamic FAs. Follow-up studies [124] provided a deeper insight into the molecular mechanisms regulating fate specification. The authors found that the creep-induced loss of cytoskeletal tension is compensated by an increase in isotonic tension with an increase of Rac1 activity, which eventually promotes myogenesis. Similar concepts have been recently developed by Chaudhuri *et al.*, who cultivated U2OS cells on either purely elastic or viscoelastic alginate hydrogels [125]. In the range of low modulus hydrogels, increased spreading and stress fiber formations were observed on stress-relaxing substrates compared to purely elastic ones. Thus, the mechanism of stress relaxation compensates for the decreased stiffness. Possibly, ligand mobility enables integrin clustering that may enhance cell spreading [112]. Taken together, these data challenge the hypothesis that mechanotransduction is solely governed by cell resistance to elastic forces, as dissipative effects can have a non-negligible role in affecting cell behavior.

**Figure 3:**
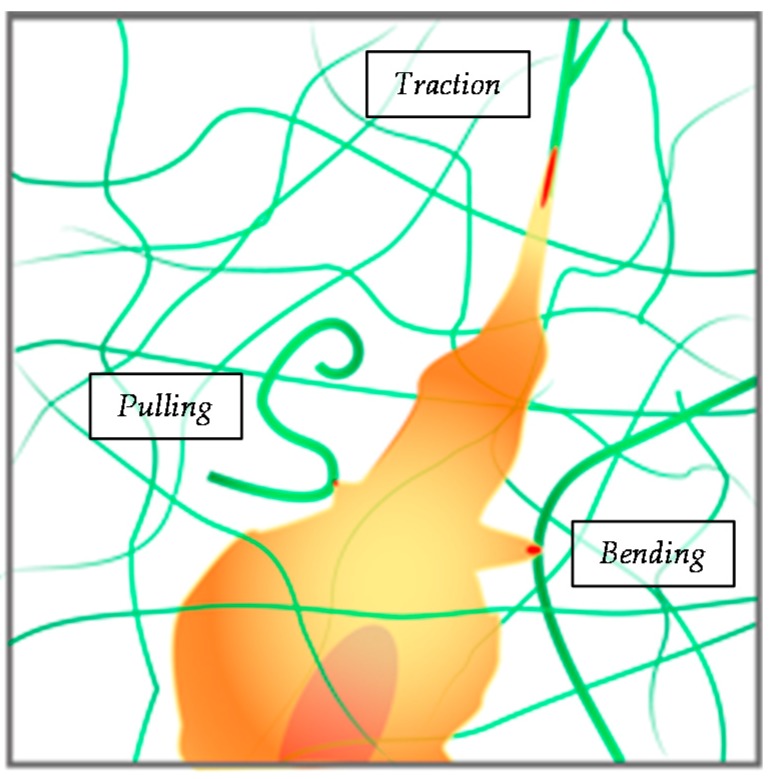
Cell perception of mechanical cues in 3D fibrillar gels. Cells might receive very different mechanical feedbacks from the surroundings according to the local configuration of the matrix. High traction forces are required for direct fibril traction, whereas low forces are necessary for fibril bending or pulling slack fibrils. Focal adhesion (FA) length and composition change accordingly.

It has to be pointed out that the examples above refer to cells cultured on hydrogels, thus representing a 2D experiment. Few characteristic traits, arising from the chemical-physical characteristics of the microenvironment, differentiate 3D mechanosensing from its 2D counterpart. Natural fibrillar gels, like collagen or fibrin gel, possess a very heterogeneous microstructure in which both long and short fibrils, along with straight and slack fibrils coexist. Therefore, a single cell might experience very different mechanical feedback according to the local network heterogeneity. For instance, individual collagen fibrils are characterized by a modulus in the range of hundreds of MPa under traction [126], whereas fibril bending or pulling buckled fibrils would produce very different mechanical feedbacks (Figure 3).

**Figure 4:**
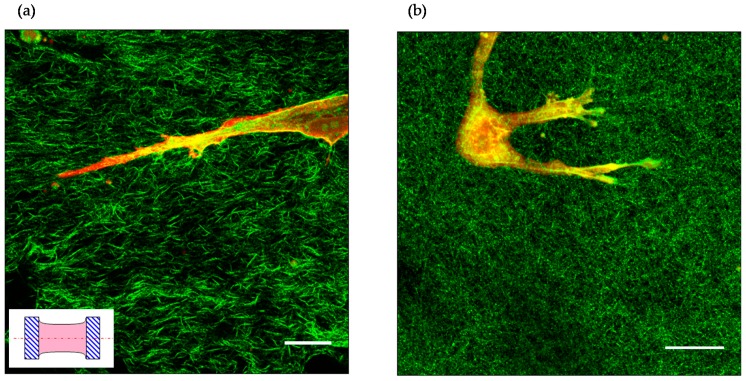
Cells encapsulated in type I collagen gel. Bovine dermal fibroblasts cultivated for one week in 2.4 mg/mL collagen gel. (**a**) In uniaxial constrained gels, cell-generated forces cause the gel to compact in the two free directions (orthogonal to the constraints; see the inset). Upon compaction, collagen fibrils result in being aligned and cells are elongated. (**b**) In free floating gels, extensive collagen densification is observed around cells, which possess a dendrite-like morphology. Collagen (green) is observed in reflection mode. Cells are stained for actin (TRITC-phalloidin). Bar = 20 µm.

Along these lines, Kubow *et al.* showed that long adhesion formed along straight fibrils, whereas small adhesions were found on angled, retracting fibrils [46]. Additionally, they reported increased amounts of vinculin and zyxin in long 3D adhesions, which is a characteristic similar to that observed in 2D. In a different experimental setup, Paszek *et al.* [127] reported reduced FAK phosphorylation at Y397 within 3D collagen gels with respect to a stiff 2D glass surface and that phosphorylation levels correlated with gel stiffness. Extensive and prolonged fibril pulling might cause dramatic network remodelling, which often results in dramatic gel compaction (Figure 4). Unconstrained gels can be compacted up to 20% of their initial volume, whereas fibril alignment is observed when gel edges are blocked, causing compaction to occur in specific planes [128,129].

This makes it hard to understand the mechanosensing mechanisms, since a local increase in fibril density, stiffness and reduced pore size occur during gel compaction. Taken together, these data suggest that while some aspects of 3D mechanosensing might find analogies with 2D setups, the intrinsic complexity of the 3D environment makes it difficult to draw out definitive conclusions on the molecular pathways governing cell recognition and reaction to mechanical stimuli, as these appear to be strongly influenced by hydrogel and cell type and experimental setups. This prompted the development of artificial systems in which stiffness and ligand availability can be varied independently. Among the various materials that have been proposed, PEG-based hydrogels have been extensively used to study mechanosensing and mechanotransduction in 3D. Other molecules, such as alginate, agarose or derivatives of hyaluronan, also offer a comparable degree of versatility in terms of manipulation of the hydrogel chemical/physical properties. The elegant study from Huebsch *et al.* clearly demonstrated how dimensionality alters the cell perception of the surrounding environment [130]. The authors encapsulated murine MSC in alginate hydrogels with stiffness varying from 2.5–110 kPa. Differently from what was observed in 2D systems on which gross cell morphological changes correlate with MSC differentiation, here, fate decisions were uncorrelated with cell morphology. Specific levels of matrix elasticity favored integrin clustering, ligand reorganization and, therefore, cytoskeletal tension, which correlated with osteogenesis. In this study, osteogenesis occurred in the case of intermediate hydrogel stiffness. Khetan *et al.* were able to induce radical crosslinking in HA-peptide hybrids that spatially confined 3D cell cultures by inhibiting degradation in specific locations [131]. This was achieved by the mutual arrangement of proteolytic crosslinks and non-degradable kinetic chains through UV exposure. Even if exposed to an osteogenic mechanical environment (~18 kPa), human MSC were unable to spread and differentiated towards an adipogenic lineage. Conversely, cells remodeled the proteolytic-sensitive domains, which allowed them to exert greater tension that favored osteogenesis despite the low stiffness of the surrounding environment. Follow-up studies also demonstrated how time changes of hydrogel stiffness could dramatically affect stem cell differentiation. Guvendiren *et al.* developed a methacrylate-functionalized HA gel that can undergo sequential crosslinking [132]. Stem cells were encapsulated in soft gels for selected time intervals, after which gels were stiffened by light irradiation. Early stiffening (*i.e*., cells exposed to a stiff environment for a long timeframe) promoted osteogenesis, whereas late stiffening promoted adipogenesis. In a more recent study, Khetan *et al.*, using HA with dynamically tunable degradation properties, investigated the mechanics governing lineage specification arising from mechanical cues in 3D [133]. Their system elegantly demonstrated that gel remodelling is essential in driving cell differentiation: even in a ‘morphologically’ spread state, cell were not able to launch an osteogenic program, owing to the low traction forces generated in a restrictive, non-degradable matrix. These examples demonstrated that matrix stiffness alone is not sufficient to predict stem cell differentiation in 3D, but it is rather the dynamic variation of stiffness and degradation that plays a crucial role in affecting cell fate.

## 5. Supramolecular Materials: Mimicking the Natural Environment of Cells

The ideal hydrogel for biomedical applications should merge the advantages of both natural and synthetic hydrogels, *i.e.*, microarchitectures similar to the native ECM and the extraordinary tunability of synthetic molecules; at the same time, such a hydrogel should minimize potential toxic effects arising from functionalization, especially when this are carried out in the presence of cells. Specifically engineered peptides forming highly hydrated nanofibrillar structures represent a very interesting class of materials that reproduces some aspect of the architecture of native ECM and allows great versatility in the modulation of the biochemical and biophysical features of the structure.

Early works by Zhang [134] showed that certain peptide sequences, inspired by natural proteins, undergo self-assembly in aqueous solutions. The peptide sequences were constituted by repetitions of hydrophilic and hydrophobic amino acids. Hydrophobic and ionic interactions between adjacent molecules cause the peptide to stack and form β-sheet structures, eventually producing nanometer-sized fibrils. Since this pioneering discovery, several peptides displaying self-assembling properties were designed. In particular, by changing the repeating amino acid sequence, it is possible to modulate hydrogel formation [135] and supramolecular organization in the form of β-hairpin or α-helices [136,137]. For example, Ramachandran *et al.* reported that mixing peptides exhibiting opposite-charged groups formed stable hydrogels that were sensitive to pH, salt and mechanical shearing forces [138]. Owing to their amenable properties of gelling in mild and cytocompatible conditions, self-assembling peptides have been widely used in Tissue Engineering applications. For instance, two formulations of peptides constituted by the repetition of arginine-alanine-aspartate (known as RAD-I and RAD-II) were used as a scaffold for neuronal regeneration. Even if these systems were not specifically functionalized with bioactive neurotropic moieties, RAD-based self-assembling hydrogels promote neuron adhesion and synapse formation [139]. Further studies were aimed at providing the gels with specific biologic functions to control cell response. Along these lines, Kumada and Zhang [140] and Kumada *et al.* [141] conjugated fibronectin- (RGD) and laminin- (PDS) derived cell adhesion sequences to RADA16-I hydrogels and reported increased fibroblast proliferation, migration and collagen biosynthesis.

MMP-sensitive domains were also incorporated into the gel building blocks in order to promote cell-mediated hydrogel degradation. Galler *et al.* engineered a self-assembling peptide hydrogel whose fibrils displayed RGD adhesion ligands and MMP2 cleavable segments [142]. These functionalities improved cell spreading and migration.

Taken together, these data demonstrate the enormous flexibility in designing peptide building blocks, thus endowing the resulting self-assembling hydrogels with biochemical/biophysical properties defined *ab initio*. Furthermore, owing to the “modular” nature of the building block structure in which individual sequences fulfil specific tasks in the self-assembling process and dictate the chemical features of the resulting network, it is in principle possible to exert an orthogonal control in engineering the final properties of the hydrogel without interfering with the spontaneous self-assembly.

Stupp’s laboratory synthesized peptide amphiphiles that self-assemble under specific conditions (pH, temperature, ionic strength). Hydrogels could be engineered in a bottom-up approach since their properties are strictly related to the structure and amount of the individual building blocks. These are constituted by four distinct regions, and the structure of each region profoundly affects the chemical/physical properties of the self-assembled hydrogel [143]. In particular, the hydrophobic sequence of the middle region bestows upon the peptide the tendency of self-assembling in the form of β-sheets. Furthermore, modifications in the structure also affect the macroscopic mechanical properties of the gel [144]. The amphiphilic nature of the peptides is provided by a hydrophobic tail and a hydrophilic peptide sequence at the other end. The latter allows good solubility and prevents self-assembly. If their charged amino acids are screened by salts or the pH of the medium is changed, self-assembly starts, eventually generating an entangled meshwork of nanofibers. Functional sequences, such as ligands or small bioactive signals, can be conjugated to the PA head, without any loss in the self-assembling capability of the system [145]. Using this strategy, specifically-designed PAs were synthesized to produce bioactive hydrogels to control cell fate and functions. Storrie *et al.* conjugated RGD sequences to PA in the form of linear, branched or cyclic geometries and studied DA 231 epithelial cell behavior *in vitro* [146]. Improved cell adhesion, spreading and migration were observed when ligands were assembled in branched, low packed structures protruding out of the fibers. This arises from an optimal presentation of adhesive signals in space. In a different setting, Silva *et al.* used the laminin-derived sequence IKVAV to control neural progenitor differentiation. IKVAV-functionalized PA gels promoted neural differentiation and suppressed astrocyte formation [147]. Interestingly, the authors also reported that IKVAV was more effective than laminin in controlling neurogenesis; this probably arises from a higher density of signals effectively presented to progenitor cells.

The above-mentioned examples demonstrated the ability of supramolecular materials to control various aspects of cellular behavior and highlighted the great potentialities these systems might have both *in vitro* and *in vivo*. However, these materials still lack some properties that would make them ideal systems to control cell behavior. First, the dynamic modulation of supramolecular material properties is still in its infancy, and only a limited number of examples of stimuli-responsive systems has been reported [148,149,150]. Second, the structures of the molecular building blocks might not make these materials directly compatible with the conventional techniques for micro- and nano-patterning. However, recent advancements in synthesizing and characterizing building blocks with enhanced functionalities and self-assembling properties will certainly help in making supramolecular materials relevant and complete platforms to control and study cell response in a chemically-/physically-controlled environment.

## 6. Future Perspectives and Conclusions

The use of engineered materials able to control and guide cell functions *in vivo* is still limited in clinical practice, in which more conservative treatments employing permanent prosthesis or resorbable devices still are the preferred choices in a large number of treatments. This notwithstanding, this review demonstrated that the studies focusing on fields of controlling cell, in particular stem cell, behavior with material surfaces or functional hydrogels are enormously active. In the future, the outcomes of these research lines might merge together to provide novel technical solutions for relevant biotechnological and clinical applications. For instance, the development of specifically-engineered materials able to exert a tight control on cell adhesion processes will be beneficial, not only to unravel the basic mechanisms governing cell reactions to exogenous stimuli, but also to fabricate complex systems or devices that control selected cell functions, like proliferation, differentiation and tissue regeneration, in a highly effective manner. Concerning *in vitro* models, the above outcomes will definitely aid the development of devices to study tissue morphogenesis and pathology progressions in a chemically-/physically-defined environment. The generation of *in vitro* models, such as organoids, although feasible, does not fully exploit the guiding effects provided by material signals. This results in substantial morphologic and functional differences between *in vitro* organoids and their counterparts found *in vivo* [151]. Yet, material features proved to profoundly affect cell self-organization and differentiation *in vitro* [152]. Similarly, the *in vivo* application of instructive scaffolds requires engineering materials displaying a set of stimuli that elicit specific cell differentiation programs and biosynthetic events in a deterministic manner. However, our current knowledge on possible relationships between material stimuli and biological responses is still in its infancy. This lack of understanding of the intricate cell-material interactions strongly limits the potential clinical applications of engineered material systems. Generally, cells integrate a combination of stimuli, also different in nature, simultaneously. This makes it difficult to deconstruct the problem, and thus, assembling combinations of stimuli to affect cell fate in a predictive manner is arduous. Owing to these difficulties, different approaches are being pursued. Combinatorial approaches, based on the fabrication of libraries of materials displaying combinations of stimuli, have been developed [153,154,155]. Such high-throughput libraries, combined with advanced investigative techniques, might help in solving the problem, thus allowing engineering systems in a deductive manner for a deterministic control of cell fate and functions.

Furthermore, because cells are naturally exposed to signals that vary in space and time according to specific programs, materials systems that enable a dynamic control of their chemical/physical properties can in principle be more effective in controlling cell functions. Few examples in which changes in properties are tailored in order to improve cell response have been recently reported in the literature [156,157].

We envision that by combining material patterning at the micro- and nano-scale and the dynamic presentation of signals with the highly tunable mechanical and biochemical properties of hydrogels will make it possible to replicate the complex environment in which cells can fulfil specific tasks defined *ab initio*. Such engineered environments will certainly impact diverse fields, both related to *in vitro* and *in vivo* investigations, such as instructive scaffolds that guide stem cell differentiation and tissue genesis for tissue engineering and regenerative medicine, the development of *in vitro* models, such as organoids or tissue analogues for drug screening and discovery, or as developmental/pathological models in an *in vitro* context.
